# TEG^®^ and ROTEM^®^ in trauma: similar test but different results?

**DOI:** 10.1186/1749-7922-7-S1-S3

**Published:** 2012-08-22

**Authors:** Ajith Sankarankutty, Bartolomeu Nascimento, Luis Teodoro da Luz, Sandro Rizoli

**Affiliations:** 1 Faculdade de Medicina de Ribeirão Preto, Universidade de São Paulo, Brazil; 2 Trauma Program, Sunnybrook Health Sciences Centre, University of Toronto, Canada; 3 Departments of Surgery and Critical Care Medicine, Sunnybrook Health Sciences Centre, University of Toronto, Canada

## Abstract

**Introduction:**

Transfusion in trauma is often empiric or based on traditional lab tests. Viscoelastic tests such as thromboelastography (TEG**^®^**) and rotational thromboelastometry (ROTEM**^®^**) have been proposed as superior to traditional lab tests. Due to the similarities between the two tests, general opinion seems to consider them equivalent with interchangeable interpretations. However, it is not clear whether the results can be similarly interpreted. This review evaluates the comparability between TEG and ROTEM and performs a descriptive review of the parameters utilized in each test in adult trauma patients.

**Methods:**

PUBMED database was reviewed using the keywords “thromboelastography” and “compare”, between 2000 and 2011. Original studies directly comparing TEG**^®^** with ROTEM**^®^** in any area were retrieved. To verify the individual test parameter used in studies involving trauma patients, we further performed a review using the keywords “thromboelastography” and “trauma” in the PUBMED database.

**Results:**

Only 4 studies directly compared TEG**^®^** with ROTEM**^®^**. One in liver transplantation found that transfusion practice could differ depending on the device in use. Another in cardiac surgery concluded that all measurements are not completely interchangeable. The third article using commercially available plasma detected clinically significant differences in the results from the two devices. The fourth one was a head-to-head comparison of the technical aspects. The 24 articles reporting the use of viscoelastic tests in trauma patients, presented considerable heterogeneity.

**Conclusion:**

Both tests are potentially useful as means to rapidly diagnose coagulopathy, guide transfusion and determine outcome in trauma patients. Differences in the activators utilized in each device limit the direct comparability. Standardization and robust clinical trials comparing the two technologies are needed before these tests can be widely recommended for clinical use in trauma.

## Introduction

Coagulation is a complex, dynamic, highly regulated and interwoven process involving a myriad of cells, molecules and structures. Only recently, the unique changes in coagulation caused by trauma are starting to be understood, but remain mostly unknown [1,2]. Trauma patients are among the largest consumers of blood and blood products and the decision of what, when and how much blood and blood product to transfuse is often empiric or based on traditional coagulation lab tests such as INR/PT, PTT and platelet count. However, traditional lab tests have been heavily criticized for their limitations in assisting the physicians with the clinical decision to transfuse, and alternatives are warranted.

The traditional laboratorial evaluation of coagulation evolved initially to quantify specific cellular, molecular or factor deficiencies. Numeric values (quantity) of individual elements do not necessarily indicate how well hemostasis is functioning. As an example, a cirrhotic patient with low platelet count and an abnormal INR of 2 does not necessarily bleed and probably can tolerate minor invasive procedures. In contrast, a hypothermic trauma patient with normal platelet count and INR might bleed to death [3,4]. Another limitation of traditional lab tests is the prolonged time to obtain the results or turnaround time. Dealing with rapid changes as frequently occurs in massively bleeding trauma patients, is challenging. In such situations, any delay in obtaining the lab results can lead to inadequate transfusion and increased morbidity and mortality [4]. Thus in trauma, global, functional and immediately available laboratorial evaluation of hemostasis can improve both patient management and outcome.

Viscoelastic tests such as thromboelastography (TEG^®^) and rotational thromboelastometry (ROTEM^®^) have been enthusiastically proposed by some, as superior compared to traditional lab tests. Both tests can be performed as point of care, and the faster availability of results may assist clinical decisions of what, when and how much blood and products to transfuse [5-7]. Other advantages of viscoelastic tests include their ability to provide a global and functional assessment of coagulation, which may prove superior to quantitative tests that evaluate segments of the hemostasis. A recent systematic review on massive transfusions concluded that despite an apparent association with bleeding reduction, the use of TEG^®^ or ROTEM^®^ to guide blood transfusion remains uncertain [8].

**Figure 1:**
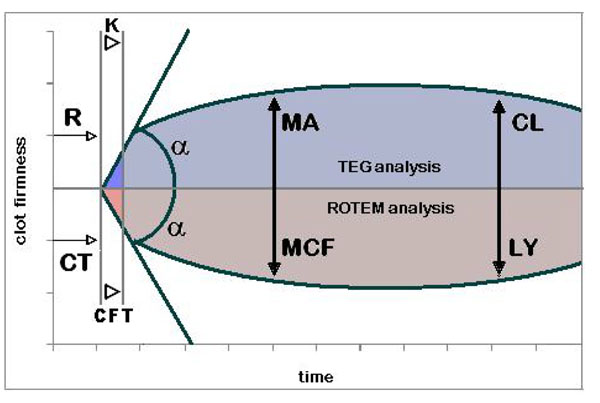
**TEG^®^ and ROTEM^®^ tracing** TEG^®^ parameters: R – reaction time; k – kinetics; ∝ - alpha angle; MA – maximum amplitude; CL – clot lysis. ROTEM^®^ parameters: CT – clotting time; CFT – clot formation time; ∝ - alpha angle; MCF – maximum clot firmness; LY – clot lysis.

The interest in TEG^®^ and ROTEM^®^ in trauma is recent and the topic lacks large numbers of studies. However, the available evidence suggests that TEG^®^ and ROTEM^®^ could have important roles in trauma in 3 ways: by promptly diagnosing early trauma coagulopathy (diagnostic tools); guiding blood transfusion and revealing patients’ prognosis. The two tests have the same foundational principles and share many similarities, from hardware (equipment) and procedures (technique) to tracing (graph) and parameters. Figure 1 merges the tracings obtained from both tests and Table 1 shows the parameters from each test and their normal values.

**Table 1:** TEG^®^ and ROTEM^®^ parameters and their reference values (adapted from Luddington 2005, and Ganter MT, Hofer CK 2008).

	TEG^®^	ROTEM^®^
Clotting time (time to 2mm amplitude)	r (reaction time) WB: 4-8min Cit, kaolin : 3-8min	CT (clotting time) Cit, EXTEM: 42-74s Cit, INTEM: 137-246s
Clot kinetics (time from 2 to 20mm)	k (kinetics) WB: 1-4min Cit, kaolin: 1-3min	CFT (clot formation time) Cit, EXTEM: 46-148s Cit, INTEM: 40-100s
Alpha angle	α (slope between r and k) WB: 47°-74° Cit, kaolin: 55°-78°	α (slope of tangent at 2mm amplitude) Cit, EXTEM: 63°-81° Cit, INTEM: 71°-82°
Amplitude (at a fixed time)	A (A30, A60)	A (A10, A15, A20, A25, A30)
Maximum strenght	MA (maximum amplitude) WB:55-73mm Cit, kaolin: 51-69	MCF (maximum clot firmness) Cit, EXTEM: 49-71mm Cit, INTEM: 52-72mm Cit, FIBTEM: 9 -25mm
Lysis (at a fixed time)	CL30, CL60	CLY30, CLY60: 94 – 100%
Maximum lysis		ML : <15%

TEG^®^ parameters: R – reaction time; k – kinetics; ∝ - alpha angle; MA – maximum amplitude; CL – clot lysis.

ROTEM^®^ parameters: CT – clotting time; CFT – clot formation time; ∝ - alpha angle;

MCF – maximum clot firmness; LY – clot lysis.

WB – whole blood; Cit – citrated blood.

The preference for which viscoelastic tests to use appears to reside primarily on geography, with centers in North America favouring TEG^®^ while Europeans prefer ROTEM^®^. Overall, the prevalent opinion is that the two tests are equivalent with interchangeable results and interpretations. It is curious to note however, that treatment recommendations seem to vary according to which test it is based on. Transfusion algorithms based on ROTEM^®^ appear to frequently recommend fibrinogen [9] while TEG^®^-based algorithms appear to recommend plasma [7]. It is not clear whether the results from these two apparently related tests are interchangeable and can be similarly interpreted. Considering the growing importance of TEG^®^ and ROTEM^®^ in trauma, attested by the growing number of viscoelastic test based algorithms and trauma centers adopting them as standard of care, we proposed a literature review on the topic. The goal is to appraise the evidence on the comparability between TEG^®^ and ROTEM^®^ as well as to perform a descriptive review of the parameters used in each test, in the setting of adult trauma patients.

## Methods

We performed a review of the literature searching PUBMED database using the keywords “thromboelastography” and “comparison”, between 2000 and 2011. Studies were eligible for inclusion if they were original and directly compared TEG^®^ with ROTEM^®^. In view of the possibility that only a small number of such studies would be found, we decided to perform an additional analysis. All studies on either TEG^®^ or ROTEM^®^ in trauma were included and each individual test parameter was scrutinized on its role in diagnosing early coagulopathy, guiding transfusion and indicating prognosis. Then the role of similar test parameters from TEG^®^ and ROTEM^®^ was compared aiming to identify whether they were comparable. For this additional analysis the review used the keywords “thromboelastography” and “trauma” in the PUBMED database. Studies were excluded if they were experimental or consisted of case reports. All full-text versions of the studies were retrieved and duplicate studies were excluded.

In this review (see Table 1), the viscoelastic test parameters will be referred to as r/CT, when referring to the initiation of the clotting process of both tests or as r when specifically referring to TEG^®^ or CT when specifically referring to ROTEM^®^. Similarly, k/CFT will refer to amplification of the clotting process, MA/MCF to the maximal clot firmness and CL/LI to fibrinolysis, in TEG^®^ and ROTEM^®^ respectively. Alpha is similar in both tests (∝).

## Results

### Direct comparison of TEG^®^ and ROTEM^®^

The literature search identified 191 studies, of which only 4 directly compared TEG^®^ with ROTEM^®^ and none were done in trauma. The two clinical studies were in liver transplantation and in cardiac surgery, another was an experiment using commercially available plasma and the last was a head-to-head comparison of the technical aspects, ease of use and costs [7,10-12]. Thus no study directly comparing TEG^®^ with ROTEM^®^ in trauma was identified. Due to the paucity of comparisons, we considered them individually.

The first clinical study by Coakley *et al*. compared transfusion triggers using TEG^®^, ROTEM^®^ (INTEM^®^ and FIBTEM^®^) and traditional coagulation tests (PT, platelet count and Clauss fibrinogen) during liver transplantation [7]. This prospective observational study showed a good correlation between TEG^®^ MA and ROTEM^®^ MCF and they shared moderate agreement in guiding platelet or fibrinogen transfusion. The study concluded that transfusion could differ depending on which device is used.

The second clinical study by Venema *et al*. compared r/CT, k/CFT, MA/MCF and the ∝ angle during cardiac surgery [10]. This study suggested that TEG® MA and ROTEM^®^ ∝ angle could be used interchangeably but the other parameters are not fully interchangeable.

The third study by Nielsen compared the reaction time, ∝ angle, maximal amplitude and maximal elastic modulus between the two devices using native plasma, celite-activated normal plasma as well as celite-activated hypo and hypercoagulable plasma [11]. All TEG^®^ ROTEM^®^ parameters were significantly different in native plasma, while in celite-activated samples most were comparable. The study concluded that the significant differences in measurements from the two devices could be attenuated with celite activation.

The head-to-head comparison of the two devices by Jackson *et al.*, took into consideration operational aspects including installation requirements, warm-up time, pipettes, material required, reference ranges, costs and opinion of the lab staff [12]. This study consisted of a simple subjective assessment of the advantages and disadvantages of both devices.

### Additional analysis of individual parameters from TEG^®^ and ROTEM^®^ in trauma

The additional PUBMED search identified 24 manuscripts, of which TEG^®^ was tested in 10, rapid-TEG in 6 and ROTEM® in 9. Two studies compared TEG^®^ with rapid-TEG^®^. No randomized controlled trial was found, 16 manuscripts analyzed data prospectively collected, 6 were retrospective and 2 were “before and after” studies. The techniques used to perform TEG^®^ and ROTEM^®^ in these 24 studies were noticeably heterogeneous. Different activators were used and different parameters evaluated making general comparisons difficult. Table 2 summarizes the main findings of the 24 manuscripts reviewed according to the test parameters evaluated and whether TEG^®^ or ROTEM^®^ were used to diagnose early coagulopathy, guide transfusion or indicate prognosis.

**Table 2:** The results and correlation of TEG^®^ and ROTEM^®^ parameters in each study for diagnosis, transfusion guidance and prognosis

Diagnosis										
**TEG**^®^	Test	Study	r /ACT	k	α	A	MA	CL	G	Comments
	
	TEG^®^	Schreiber (2005)	PTT				Platelet			
		
		Johansson (2008b)								r, k, α, MA and G improved after Tx packages
		
		Plotkin (2008)					Platelet			
		
		Park (2009)			NO correlation to PT/PTT		NO correlation to PT/PTT			
		
		Watters (2010)								MA significantly higher post-splenectomy
	
										
	
	TEG^®^-PM	Nekludov (2007)								Reduced platelet response to AA in bleeders
	
										
	
	Rapid-TEG^®^	Jeger (2009)		Platelet/INR	Platelet/INR		Platelet/INR			
		
		Cotton (2011)	PT/PTT	PT/PTT	PT/PTT/platelet		PT/PTT/platelet		No correlation	

										

**ROTEM**^®^	Test	Study	CT	CFT	α	CA	MCF	CLI	ML	
	
	EXTEM^®^	Rugeri (2006)				PT (CA15)				
		
		Levrat (2008)				ELT (CA10)	ELT	ELT (CLI60)		
		
		Davenport (2011a)								CT, CA, MCF improves after Tx
		
		Davenport (2011b)								CA5 diagnosis coagulopathy
	
										
	
	INTEM^®^	Rugeri (2006)		PTT		PTT / Platelet (CA15)				
	
										
	
	FIBTEM^®^	Rugeri (2006)				Fibrinogen (CA10)				

										

**Transfusion Guidance**										

**TEG**^®^	Test	Study	r / ACT	k	α	A	MA	CL	G	Comments
	
	Rapid-TEG^®^	Kashuk (2009)	Could reduces FFP Tx							

										

**ROTEM**^®^	Test	Study	CT	CFT	α	CA	MCF	CLI	ML	
	
	EXTEM^®^	Schochl (2011)								ROTEM guided FC/PCC reduces RBC and platelet Tx
	
										
	
	FIBTEM^®^	Schochl (2011)								ROTEM guided FC/PCC reduces RBC and platelet Tx

**Prognosis**										

**TEG**^®^	Test	Study	r / ACT	k	α	A	MA	CL	G	Comments
	
	TEG^®^	Plotkin (2008)					Increased Tx			
		
		Park (2008)					Mortality			
		
		Johansson (2008a)								TEG guided Tx reduced mortality
		
		Carroll (2009)	Mortality				Mortality			
	
										
	
	TEG^®^-PM	Carroll (2009)								Significantly correlated to Tx
	
										
	
	Rapid-TEG^®^	Kashuk (2010)							Mortality	
		
		Kashuk (2012)						Mortality	Mortality	
		
		Pezold (2012)							Massive Tx; Mortality	

										

**ROTEM**^®^	Test	Study	CT	CFT	α	CA	MCF	CLI	ML	
	
	EXTEM^®^	Schochl (2009)							Mortality	
		
		Doran (2010)					Increased Tx			
		
		Schochl (2010)								ROTEM guided Tx reduces mortality
	
										
	
	INTEM^®^	Leemann (2010)					Increased Tx			

Abbreviations: r, k, α and MA – TEG^®^ parameters; CT,CFT, α, MCF, CA_10_, CA_15_, CL_30_-CL_60 –_ ROTEM^®^ parameters; ACT – activated clotting time; ELT – euglobulin lysis time; FFP – fresh frozen plasma; G – maximal elastic modulus (d/sc); PC – platelet concentrate; PCC – prothrombin complex concentrate; Tx – transfusion

### Results of 12 studies on the use of TEG^®^ or ROTEM^®^ as diagnostics tools

Among the studies on TEG^®^ Schreiber *et al* reported a correlation between r and PTT, and between MA and platelet count [13]. While Plotkin *et al* found a similar correlation between MA and platelet [14], Jeger *et al* found that k, ∝ angle and MA correlated with platelet levels and INR [15]. Park *et al* found no correlation between either ∝ angle or MA to PT and PTT [16] while Cotton *et al* (using Rapid TEG) reported a correlation between ∝ angle and MA with platelet, PT and PTT. In this study G was failed to correlate with any traditional lab tests [17].

Johansson *et al* reported that all the TEG^®^ parameters improved after the administration of predefined transfusion packages [18]. Watters *et al* reported that MA parameters were higher in patients after splenectomy [19]. Using the platelet mapping sequence in the TEG^®^, Nekludov found that bleeding patients have reduced platelet response to arachdonic acid [20].

In ROTEM^®^ studies Rugeri found that CA15-EXTEM^®^ correlated with PT, CA15-INTEM^®^ with platelets and PTT, and CA10-FIBTEM^®^ with fibrinogen [21]. Levrat *et al* noted that in EXTEM^®^ CA10, MCF and CLI60 correlated well with the euglobulin lysis time, which they used as the gold standard to detect fibrinolysis [22]. Davenport *et al* reported that CA5 could be an early indicator of coagulopathy in trauma and CT, CA and MCF improved after transfusion [23,24].

In summary, the single apparent similarity between TEG^®^ and ROTEM^®^ parameters when used to diagnose coagulopathy in trauma is between TEG^®^ MA and ROTEM^®^ MCF and their similar association to platelet count and PTT.

### Results of the 2 studies on the use of TEG^®^ and ROTEM^®^ in guiding transfusion in trauma

In a retrospective study, Kashuk *et al* suggested that using TEG^®^ parameters such as r to guide transfusion may lead to a reduction in plasma transfusion [25]. Schochl *et al* reported that ROTEM^®^-based protocols are useful to guide transfusion of fibrinogen concentrates and prothrombin complex that in turn reduce the need for transfusion of red blood cells and platelets [26]. As summarized in Table 2, no similarity between TEG^®^ and ROTEM^®^ can be made from these studies.

### Results of the 11 studies on the use of TEG^®^ and ROTEM^®^ and outcome in trauma

Plotkin *et al* in a retrospective study on TEG^®^ reported that low MA correlated with increased transfusion requirement [14]. For ROTEM^®^, 2 studies by Leeman *et al and* Doran *et al* reported the same finding with MCF (INTEM^®^), the later study also showed that reduced MCF (EXTEM^®^) is useful to guide transfusion [27,28].

Park developed a prognostic scoring system for trauma patients using inflammatory and coagulation parameters, in which of all TEG^®^ parameters only MA was an independent predictor of mortality [29]. Carroll also detected a significant correlation between TEG^®^ platelet mapping and transfusion requirements, and a correlation between r and MA values with mortality [30]. Kashuk in both a “before and after” and a prospective observational study found that TEG^®^ G values were associated with survival [31]. Similarly Pezold in a retrospective TEG^®^ study found that low G values were associated with both increased transfusion requirements and mortality [32]. Both, Johansson (“before and after” TEG^®^ study) and Schochl (ROTEM^®^ retrospective study) suggested that viscoelastic tests guided transfusion reduced mortality [5,9]. Schochl also reported that hyperfibrinolysis, detected by ROTEM^®^ ML correlated with higher mortality and this parameter could be used to classify the degree of severity of the fibrinolysis [33]. In 2010 Kashuk et al found that abnormal primary lysis detected by elevated CL (similar to ROTEM^®^ ML) is also associated with mortality [31].

As summarized on Table 2, these 11 studies showed that some TEG^®^ and ROTEM^®^ parameters are similarly associated with outcomes in trauma. TEG^®^ MA and ROTEM^®^ MCF are associated with both the need for blood transfusion and mortality, while excessive fibrinolysis diagnosed by either TEG^®^ CL or ROTEM^®^ ML are independent predictors of mortality.

## Discussion

A few deductions can be promptly reached from reviewing the literature on these two viscoelastic tests. First that there is a lot of enthusiasm supporting their clinical application in trauma. The literature suggests that both tests are already being used in many institutions, which could be in a wider scale than suggested by the limited number of publications. The wide clinical application of any technology without supporting evidence and scientific validation is worrisome and more investigations on these tests are urgently needed and warranted.

Another plausible conclusion from this review is that the prevalent notion that the two tests are equivalent with interchangeable results and interpretations may be unfounded. While there are insufficient studies to support any conclusions on the topic, the current evidence indicates only a small number of similarities between the tests. Concerning their diagnostic capacity, the similarities found were limited to TEG^®^ MA and ROTEM^®^ MCF and their similar association with platelet count and PTT. Another apparent similarity was of TEG^®^ CL and ROTEM^®^ ML in diagnosing excessive fibrinolysis and mortality (prognosis). Prognostication was where these tests showed more similarities. TEG® MA and ROTEM MCF® were also linked to the need for blood transfusion and mortality. The few studies on TEG^®^- or ROTEM^®^-based transfusion algorithms suggested that while both tests can be used to construct transfusion guidelines, the blood products transfused differ according to the algorithm selected.

Even tough no study could be found directly comparing TEG^®^ and ROTEM^®^ in trauma; two studies have compared the 2 tests in transplant and cardiac surgery. Coakley *et al.*, in the liver transplant study concluded that transfusion practice could differ depending on the visco-elastic coagulation-monitoring device in use. Venema *et al.*, verified that kaolin-activated TEG^®^ measurements correlated with those of EXTEM^®^, but not all the measurements of the two devices are interchangeable. These findings seem to support the concept that despite similarities, interchangeable interpretation is not recommended without further studies and standardizations.

Despite being used for a number of years, the recent wider adoption and transfer of the technology to the hemostasis laboratory has raised some concerns regarding these techniques. Among the concerns pointed out in the literature are the effect of age [34-37], gender [38], use of citrated blood sample [39], sampling site, stability and repeated sampling [40-43] on the results observed. A number of activators and inhibitors are commonly used resulting in varied specificity of the assay [44]. Different methods of data analysis have also been suggested [45]. In an interesting article Jackson *et al* “road tested” both TEG^®^ and ROTEM^®^ and summarized their finding regarding technical features, costs and pooled the opinion of the direct users [12]. The reproducibility of both TEG^®^ and ROTEM^®^ measurements has been reported as acceptable [46].

A recent systematic review of randomized clinical trials comparing TEG^®^- or ROTEM^®^-based algorithms with standard treatment in non-trauma bleeding patients found that the current evidence supporting viscoelastic tests is weak [4]. This systematic review found only 9 randomized controlled trials, 8 in cardiac surgery and 1 in liver transplantation. Possibly the greatest contribution of the viscoelastic tests is in the detection of hyperfibrinolysis, which no other test can diagnose as expeditiously.

Interestingly, Nielsen pointed out in his study that TEG^®^ and ROTEM^®^ could potentially generate similar data, provided similar activators were utilized in both devices. This observation highlights the need for standardization if the tests are to be comparable. Meanwhile, caution must be exercised in utilizing treatment algorithms based on one system while analyzing patient samples from the other, or even the same system but using different activators.

In conclusion, TEG^®^ and ROTEM^®^ have many of the characteristics of ideal tests for use in trauma including global evaluation of coagulation, both quantitative and functional assessment, *in vitro* assays performed under conditions of ”no flow”. Their potential clinical utility must be balanced against limitations particularly the considerable heterogeneity in methods, reagents and parameters evaluated. The present literature review suggests that in trauma TEG^®^ and ROTEM^®^ are not fully equivalent tests with interchangeable results and interpretations but as pointed out by Nielsen, this could be the results of using different activators (methods). The similarities identified were limited to TEG^®^ MA and ROTEM^®^ MCF measurements and their association with platelet counts and PTT. Other similarities were between TEG^®^ CL and ROTEM^®^ ML in diagnosing excessive fibrinolysis and mortality and TEG^®^ MA and ROTEM^®^ MCF association with blood transfusion and mortality.

Despite their limitations, both tests are attractive and potentially useful as means to rapidly diagnose coagulopathy, guide transfusion and determine outcome of adult trauma patients. However, standardization and robust clinical trials comparing the two technologies are needed before these promising tests can be widely recommended for clinical use in trauma.

## Authors' contributions

Literature review and drafting the manuscript : AS, LTdL, BN

Drafting the manuscript and critical review: SR

## Competing interests

SR had a Canadian Institutes of Health Research (CIHR) award in partnership with NovoNordisk the manufacturer of recombinant factor VIIa.

The other authors declare that they have no competing interests.
